# Supplementary material for: Hypoosmotic stress induces flagellar biosynthesis and swimming motility in Escherichia albertii
Source: Commun Biol. 2020 Feb 28;3:87. doi: 10.1038/s42003-020-0816-5 (PMC7048735; doi:10.1038/s42003-020-0816-5)
Supplement: Supplementary file 2 — Descriptions of Additional Supplementary Files [file 42003_2020_816_MOESM2_ESM.pdf]

Descriptions of additional supplementary files

Supplementary Movie 1
Swimming cells of *E. albertii* (HIPH16576) imaged by DIC.

Supplementary Data 1
Source data for graphs presented in Figure 1, 2, and 3.
