# Supplementary material for: Hypoosmotic stress induces flagellar biosynthesis and swimming motility in Escherichia albertii
Source: Commun Biol. 2020 Feb 28;3:87. doi: 10.1038/s42003-020-0816-5 (PMC7048735; doi:10.1038/s42003-020-0816-5)
Supplement: Supplementary file 3 — Supplementary Tables [file 42003_2020_816_MOESM3_ESM.pdf]

Supplementary Table 1: A list of *E. albertii* subjected to whole genome sequencing in this study

Strain	Pathogenic gene	Source	Accession number (ref.)
HIPH08472	*eae, stx2f, astA*	Human patient	BBVZ01000001-BBVZ01000085 (1)
HIPH12313	*eae, stx2f*	*Pica pica*	BLJJ01000001-BLJJ01000253 (this work)
HIPH12338	*eae*	*Larus schistisagus*	BJWR01000001-BJWR01000369 (2)
HIPH14005	*eae*	*Corvus macrorhynchos*	BLJK01000001-BLJK01000231 (this work)
HIPH14564	*eae*	*Corvus corone*	BLJL01000001-BLJL01000353 (this work)
HIPH16576	*eae, stx2f*	*Corvus macrorhynchos*	BLJM01000001-BLJM01000228 (this work)

Supplementary Table 2: Oligonucleotide primes for qRT-PCR

flhD	F: TCTTGCACAACGTCTGATCG<br>R: CGGGTAATAGTCTGGTGGCT
fliD	F: CACCACCAGAGACGATACGA<br>R: GCCGGTGTCATTTGATGTGA
16S rRNA	F: GCGGTGAAATGCGTAGAGAT<br>R: CGTTGCATCGAATTAAACCA

Supplementary References

1. Ooka, T. *et al*. Defining the Genome Features of *Escherichia albertii*, an Emerging Enteropathogen Closely Related to *Escherichia coli*. *Genome Biol Evol* **7**, 3170–3179 (2015).
2. Ooka, T. *et al*. O-antigen biosynthesis gene clusters of *Escherichia albertii*: their diversity and similarity to *Escherichia coli* gene clusters and the development of an O-genotyping method. *Microb Genom* **5**, (2019).
